# The impact of loneliness on self-rated health symptoms among victimized school children

**DOI:** 10.1186/1753-2000-6-20

**Published:** 2012-05-29

**Authors:** Audhild Løhre

**Affiliations:** 1 Research Centre for Health Promotion and Resources HiST/NTNU, Department of Social Work and Health Sciences, Norwegian University of Science and Technology, Trondheim, Norway; 2 Department of Public Health, Faculty of Medicine, Norwegian University of Science and Technology, Trondheim, Norway

## Abstract

**Background:**

Loneliness is associated with peer victimization, and the two adverse experiences are both related to ill health in childhood and adolescence. There is, however, a lack of knowledge on the importance of loneliness among victimized children. Therefore, possible modifying effects of loneliness on victimized school children’s self-rated health were assessed.

**Methods:**

A population based cross-section study included 419 children in grades 1–10 from five schools. The prevalence of loneliness and victimization across grades was analyzed by linear test for trend, and associations of the adverse experiences with four health symptoms (sadness, anxiety, stomach ache, and headache) were estimated by logistic regression.

**Results:**

In crude regression analysis, both victimization and loneliness showed positive associations with all the four health symptoms. However, in multivariable analysis, the associations of victimization with health symptoms were fully attenuated except for headache. In contrast, loneliness retained about the same strength of associations in the multivariable analysis as in the crude analysis. More detailed analyses demonstrated that children who reported both victimization and loneliness had three to seven times higher prevalence of health symptoms compared to children who reported neither victimization nor loneliness (the reference group). Rather surprisingly, victimized children who reported no loneliness did not have any higher prevalence of health symptoms than the reference group, whereas lonely children without experiences of victimization had almost the same prevalence of health symptoms (except for stomach ache) as children who were both victimized and lonely.

**Conclusions:**

Adverse effects of loneliness need to be highlighted, and for victimized children, experiences of loneliness may be an especially harsh risk factor related to ill health.

## Background

Despite well documented associations of peer victimization with loneliness [1-4] health related effects of loneliness among victimized children have not been extensively studied [5]. Loneliness is a hurtful feeling [6,7] that has been attributed to a discrepancy between desired and achieved levels of social contact [8]. Most children have an intuitive understanding of loneliness [9,10], and both being alone and sadness are included in their understanding [9].

The results of many studies have suggested that loneliness is associated both with anxiety and depression among children [1,11-14]. Also, lonely children appear to be less accepted [15] and more rejected by their peers [9,16-18]. Compared to popular children who have many friends, the lonely children have fewer, and children with no friends appear as the most lonely [16,19]. Intervention studies that aimed to increase the students’ attachment or belongingness to their school have shown reduced loneliness among the participants [20,21].

Victimization (being bullied) is a harsh form of peer rejection, and includes being the target of aggressive behaviour, repetitive negative acts and imbalance of power [22-24]. There is consensus that children who are subject to bullying are at increased risk of mental health problems [25,26], psychosomatic illness [27], and psychosocial maladjustments [28,29]. Further, it has been suggested that rejected, anxious, or depressed children in the next turn more easily are bullied by peers than children without internalizing or adjustment problems [30,31].

Previous studies have shown that friendship among peers may modify and protect against the adverse effects of victimization [32,33]. Whether loneliness also has a modifying effect, has scarcely been studied. Therefore, we have used population data among school children to assess whether the health related effects of victimization could be modified by loneliness. We hypothesized that children who report both victimization and loneliness would have a higher prevalence of health symptoms than victimized children who do not report loneliness.

## Methods

### Participants and procedure

This study is based on cross-sectional data from children in a convenience sample of five schools in Møre and Romsdal County, Norway. Three schools had grades from 1 to 7, and two schools had grades from 1 to 10. All children from four schools and all children in grades 7–10 from the fifth school were included. In total, 423 children between seven and 16 years of age were invited. One child moved before the data collection started, and three children were on sick leave during the study period. Thus, 419 (99%) children participated in the study.

Parents were informed about the survey in the context of a school meeting, and in each class, teachers informed the children about the survey. Information letters signed by the headmaster and by the principal investigator (AL) were sent to all parents, describing the aims of the survey, and emphasising that participation was voluntary and that the collected information was confidential. Children/parents who did not want to participate were asked to notify their main teacher or headmaster. None of the subjects declined to take part in the survey.

The collection of data was administered by school nurses and headmasters, and all children answered the School wellbeing – Student questionnaire [34]. Most of the informants filled in the questionnaire themselves, but younger children and children who had problems with reading or writing were interviewed by the school nurses. Thus, 180 children in grades 1- 4, 53 children in grades 5–7, and three children in grades 8–10 were interviewed by trained school nurses who used the questionnaire as a guide. Under the instruction of the school nurse or a trained teacher, the remaining 183 children completed the questionnaires themselves during a lesson that was allocated to this task.

### Measures

The School wellbeing – Student questionnaire has demonstrated satisfactory construct, content, and face validity, as described in detail elsewhere [34,35]. Briefly, the questionnaire consists of a combination of items that potentially may promote school wellbeing or health, and items that may be adversely associated with school wellbeing or health. Responses to the questions are ranked on ordinal scales, with four or five response options. Some of the items addressed in the questionnaire are more relevant for experiences during lessons and some items are more relevant in recess.

Reliability of the School wellbeing – Student questionnaire was tested in another material gathered from children in grades 3, 6, and 9. Among 179 eligible children, the questionnaire was completed two times, three weeks apart, by 154 (86%) children. The test- retest reliability for the variables used in the present study was acceptable: the correlation coefficients varied from 0.46 to 0.57 (all p-values <0.001).

Responses to the questions were to be relevant for the current school year, and responses were ranked on ordinal scales. The following items were addressed, each with the corresponding questions:

Loneliness. One question was asked: “Do you ever feel lonely at school?” with five response options (1–5): never, seldom, sometimes, about every week, and about every day.

Victimization. Three questions were asked: “During recess, are you bothered in some way that makes you feel bad: 1) by being teased, 2) by being hit, kicked, or pushed, 3) by being left out, excluded?”. Each question had five response options (1–5): never, seldom, sometimes, about every week, and about every day. In the analyses, we employed the question(s) with the highest response score of the three questions (the max score, i.e. one score only).

In some of the analysis, victimization and loneliness were dichotomized into never/seldom (defined as no victimization or no loneliness, respectively) versus sometimes/weekly/daily (defined as victimization or loneliness, respectively). Further, four groups of children were composed: children who reported both victimization and loneliness (denoted Victim-and-lonely); children who reported victimization but not loneliness (denoted Victim-not-lonely); children who reported loneliness but not victimization (denoted Lonely-not-victim); and those who reported neither victimization nor loneliness (denoted Not-lonely-not-victim).

Health symptoms. Four questions were asked: Each question had five response options (1–5): never, seldom, sometimes, often, and always. Sadness and anxiety were denoted internalizing symptoms, and stomach ache and headache were denoted somatic symptoms. In the analyses, each health symptom (as an outcome) was dichotomized into never/seldom versus sometimes/ often/always.

### Ethics

The survey was approved by the statutory School Collaborative Committees, and the collection of data was approved by The Norwegian Data Inspectorate.

### Statistics

Differences in frequencies of victimization and loneliness across school grades were analyzed by a linear test for trend, and logistic regression analysis was used to assess the associations of victimization and loneliness with the odds of reporting health symptoms. Precision of the associations (odds ratios (OR)) was assessed by 95% confidence intervals. Tests for statistical significance were two-sided, and p-values < 0.05 were considered significant. The statistical analyses were performed in SPSS for Windows (version 18 SPSS, Chicago, Illinois).

## Results

Among the 419 children, 20.6% had experienced victimization sometimes, weekly, or daily, and 17.9% reported the same frequency of loneliness (Table 1, options 3–5). Further, approximately one fourth of the children reported sadness, stomach ache, or headache sometimes, often, or always (options 3–5), and less than one in five had experienced a corresponding frequency of anxiety.

**Table 1:** **Distribution of response options for dependent**^1^**and independent**^2^**variables in a population study of Norwegian school children**

**Response options**^**a**^								
	**1**	**2**	**3**	**4**	**5**	**Total**		
**Variables**	**%**	**%**	**%**	**%**	**%**	**N**	**Median**	IQR*
Sadness ^1^	24.5	48.9	23.5	2.7	0.5	413	2	2-3
Anxiety ^1^	54.7	28.0	12.9	3.2	1.2	411	1	1-2
Stomach ache ^1^	39.6	31.9	21.7	5.1	1.7	414	2	1-3
Headache ^1^	38.7	28.5	23.6	7.3	1.9	411	2	1-3
Loneliness ^2^	60.5	21.5	14.8	1.4	1.7	418	1	1-2
Victimization ^2^	55.2	24.2	16.5	2.2	1.9	417	1	1-2

a From 1 (best) to 5 (worst).

* 25-75th percentile.

Of the 86 children who reported to be victimized sometimes, weekly, or daily, half of them reported never or seldom being lonely and the other half reported being lonely sometimes, weekly, or daily (Table 2). Among the first half, 3 (7.0%) were victimized weekly or daily, and among the second half, 14 (32.6%) were victimized weekly or daily.

**Table 2:** Cross-table of loneliness and victimization in a population study of Norwegian school children

**Loneliness**						
**Victimization**	**Never**	**Seldom**	**Sometimes**	**Weekly**	**Daily**	**Total**
Never	184	30	15	1	0	230
Seldom	46	38	16	0	0	100
Sometimes	21	19	24	2	3	69
Weekly	1	0	4	3	1	9
Daily	0	2	3	0	3	8
Total	252	89	62	6	7	416

In crude analyses adjusting for gender and grade, both loneliness and victimization showed significant associations with each of the four health symptoms (left side of Tables 3). However, in the multivariable analysis, the associations of victimization with sadness, anxiety, and stomach ache were fully attenuated. On the other hand, corresponding associations of loneliness were hardly changed (right side of Table 3). Loneliness demonstrated significant associations with sadness (odds ratio, 1.7, 95% CI 1.3 to 2.3), anxiety (odds ratio, 2.3, 95% CI 1.7 to 3.2), and stomach ache (odds ratio, 1.4, 95% CI 1.1 to 1.9). In relation to headache (right side of Table 3), loneliness and victimization showed approximately the same strength of associations (odds ratio, 1.3, 95% CI 1.0 to 1.8 and odds ratio, 1.4, 95% CI 1.0 to 1.8, respectively).

**Tables 3:** a-d, Associations (Odds ratio, 95% CI) of loneliness and victimization with self-reported health symptoms in a population study of Norwegian school children

	Each covariate* adjusted only for gender and grade		**Loneliness, victimization, gender and grade included in the model**	
	**Odds ratio**		**Odds ratio**	
	Estimat (95% CI)	p-value	Estimat (95% CI)	p-value
**a. Sadness**				
Loneliness	1.8 (1.4 to 2.2)	<0.001	1.7 (1.3 to 2.3)	<0.001
Victimization	1.4 (1.1 to 1.7)	0.006	1.0 (0.8 to 1.4)	0.799
**b. Anxiety**				
Loneliness	2.4 (1.8 to 3.2)	<0.001	2.3 (1.7 to 3.2)	<0.001
Victimization	1.7 (1.3 to 2.2)	<0.001	1.1 (0.8 to 1.5)	0.581
**c. Stomach ache**				
Loneliness	1.6 (1.3 to 2.1)	<0.001	1.4 (1.1 to 1.9)	0.016
Victimization	1.5 (1.2 to 1.9)	<0.001	1.3 (1.0 to 1.7)	0.074
**d. Headache**				
Loneliness	1.6 (1.2 to2.0)	<0.001	1.3 (1.0 to 1.8)	0.046
Victimization	1.6 (1.3 to 2.0)	<0.001	1.4 (1.0 to 1.8)	0.025

* Loneliness and victimization.

Separate analyses for boys and girls did not demonstrate any substantial differences between the genders in the multivariable analyses, except for headache. For boys, both victimization and loneliness showed statistically non-significant associations with headache, but for girls, loneliness was related to headache (odds ratio, 1.5, 95% CI 1.0 to 2.3) whereas victimization showed a weaker association.

Loneliness and victimization were further explored in the groups of children defined by combinations of loneliness and victimization. Approximately seven in ten children reported no loneliness and no victimization, two in ten had experienced victimization (one in combination with loneliness and one without), and less than one in ten reported loneliness without being victimized (Table 4). More children were victimized during the earlier years in school compared to later years. A significant downward trend was shown both for the Victim- not-lonely group (p = 0.001) and the Victim-and-lonely group (p = 0.022) whereas the Lonely-not-victim group had no increase or decrease across the school grades (p = 0.240).

**Table 4:** Number and percentage of children across grades by combinations of loneliness and victimization in a population study of Norwegian school children

	**Not-lonely- not-victim**		**Lonely-not- victim**		**Victim-not- lonely**		**Victim-and- lonely**		**Total (100%)**
Grades	N	(%)	N	(%)	N	(%)	N	(%)	N
1	19	59.4	4	12.5	4	12.5	5	15.6	32
2	28	53.8	6	11.5	9	17.3	9	17.3	52
3	33	64.7	5	9.8	7	137	6	11.8	51
4	31	68.9	2	4.4	9	20.0	3	6.7	45
5	28	68.3	2	4.9	4	9.8	7	17.1	41
6	32	78.0	2	4.9	4	9.8	3	7.3	41
7	36	78.3	4	8.7	3	6.5	3	6.5	46
8	29	82.9	3	8.6	0	0.0	3	8.6	35
9	27	96.4	0	0.0	0	0.0	1	3.6	28
10	35	77.8	4	8.9	3	6.7	3	6.7	45
Total	298	71.6	32	7.7	43	10.3	43	10.3	416

Lonely-not-victim – Linear by linear test for trend: p = 0.240.

Victim-not-lonely – Linear by linear test for trend: p = 0.001.

Victim-and-lonely – Linear by linear test for trend: p = 0.022.

After adjusting for gender and grade (Table 5), the Victim-and-lonely group showed the highest prevalence of health symptoms and was strongly and positively associated with sadness (odds ratio, 3.8, 95% CI 1.9 to 7.6), anxiety (odds ratio, 6.5, 95% CI 3.1 to 13.7), stomach ache (odds ratio, 3.4, 95% CI 1.7 to 6.6), and headache (odds ratio, 3.4, 95% CI 1.7 to 6.8). Except for stomach ache, the Lonely-not-victim group showed nearly the same strength of associations: sadness (odds ratio, 3.3, 95% CI 1.6 to 7.2), anxiety (odds ratio, 5.6, 95% CI 2.4 to 12.8), headache (odds ratio, 2.6, 95% CI 1.2 to 5.7). On the other hand, the Victim-not-lonely group showed no significant associations with the health symptoms.

**Table 5:** Combinations of loneliness and victimization associated (Odds ratio, 95% CI) with self-reported health symptoms in a population study of Norwegian school children

	**Sadness**	**Anxiety**	**Stomach ache**	**Headache**
Covariate*	**OR (95% CI)**	**OR (95% CI)**	**OR (95% CI)**	**OR (95% CI)**
Not-lonely-not-victim	Ref.	Ref.	Ref.	Ref.
Lonely-not-victim	3.3 (1.6 to 7.2)	5.6 (2.4 to 12.8)	1.9 (0.9 to 4.3)	2.6 (1.2 to 5.7)
Victim-not-lonely	1.3 (0.6 to 2.8)	2.1 (0.9 to 5.0)	1.7 (0.9 to 3.5)	1.4 (0.7 to 3.0)
Victim-and-lonely	3.8 (1.9 to 7.6)	6.5 (3.1 to 13.7)	3.4 (1.7 to 6.6)	3.4 (1.7 to 6.8)

* Categorical covariate adjusted for gender and grade in binary logistic regression.

## Discussion

In cross-sectional data on 419 school children, the impact of loneliness among victimized children was assessed in relation to self-reported health symptoms. The main finding was the adverse and modifying influence of loneliness among victimized children. As hypothesized, victimized children who also felt lonely had higher prevalence of health symptoms than victimized peers who did not report loneliness. The unexpected finding, however, was the large gap in prevalence of health symptoms between the two groups. Children who experienced both loneliness and victimization had three to seven times higher prevalence of internalizing or somatic symptoms compared to children who reported no victimization and no loneliness (the reference group). In contrast, victimized children who reported no loneliness were no more likely to have any health symptoms than the reference group. Moreover, lonely children who reported no victimization, showed approximately the same prevalence of internalizing symptoms and headache as lonely children who were victimized.

The study was conducted in rural communities, ranging from inland to coastal areas. All children attended schools in the Norwegian public school system. The population base and the very high participation are strengths of the study, but it is a weakness that the data do not include children from urban settings. The convenience sampling of schools may also be a limitation. The reported prevalence of victimization and the decline across school grades are, however, in line with results from other relevant studies [24,36-39] and may support the external validity of the findings. By carefully following the questionnaire, school nurses interviewed the youngest children, whereas older children completed the questionnaire themselves. Although the nurses were trained for this task, we cannot exclude the possibility that different procedures could have influenced the collected information and introduced systematic differences in results between younger and older children. As described in the methods the variables applied in the study were dichotomized never/seldom versus sometimes or more often. The chosen cutpoint was preferable as regards the dispersion of data (Table 1) and power of the analyses, and also it was important to have a reference group of children who were neither victimized nor lonely. The cross-sectional design is a limitation of this study. Compared to longitudinal designs that allow investigating causal effects the cross-sectional design limit the researcher to report on associations. The findings must therefore be interpreted with caution.

It may be argued that the Victim-not-lonely group of children had lower proportions of weekly or daily victimization than the Victim-and-lonely group, and this may explain some of the differences between the two groups as higher frequencies of victimization are known to have stronger associations with health symptoms than lower frequencies [40-42]. On the other hand, victimization at lower frequency, i.e. sometimes, has also shown strong and consistent associations with health symptoms [41,42]. One marked difference between those studies and the present study is, however, the exclusion of lonely children in the Victim-not-lonely group. Further, lonely children had a high prevalence of health symptoms regardless of reporting victimization or not. This finding is rather surprising since an additive effect of victimization could be expected for the lonely children who were also victimized. Possibly, the finding reflects loneliness as unique and painful experiences with strong individual relations to health symptoms. This is in line with research that presents strong associations of loneliness with depression and anxiety [11,13,43]. The relation between loneliness and somatic symptoms are, however, scarcely explored.

The two groups Victim-and-lonely and Victim-not-lonely showed a decrease across school grades, and these findings correspond to previous results on victimization by bullying that have reported far more children to be victimized during the first years in school than in later years [36,37]. For the Lonely-not-victim group, another pattern was revealed with no significant downward or upward trends across the ten school years. To our knowledge, no other studies have reported measures on loneliness across ten school grades (from 7 to 16 years of age), but our findings are supported by publications that have reported approximately the same prevalence of loneliness in US children from preschool to sixth grade [9,16]. On the other hand, Greek and Finnish studies have reported a remarkably higher prevalence of loneliness among primary school children [10,44,45].

The strong association of loneliness with internalizing as well as somatic symptoms calls for attention in schools and health care. It is important to search for strategies that prevent the development of loneliness and additionally, protect against painful feelings related to loneliness. Findings in previous studies may lead to some suggestions of strategies including friendship, participation in class, and belongingness to school. After training of the students’ social and emotional skills intended to increase the students’ belongingness to school, the prevalence of loneliness among students was reduced [20,21] and also, belongingness to school may be a buffer against depression among lonely children [46]. Further, loneliness may be related to shyness and indirectly to passivity [47] and it has been suggested to work with participation in the classroom instead of working directly with loneliness [47]. This strategy may be supported by a study reporting negative associations of loneliness with competence and support from peers [48]. Thus, circles of passivity, sadness, rejection, and isolation can be turned to positive loops of participation, skilled interactions, increased popularity, and more friends [19-21,47]. Furthermore, training of social and emotional skills and participation in the classroom can be included in the daily activities at school, but there is a need for studies designed to evaluate such pedagogical practice.

## Conclusions

Our findings indicate that some children who report victimization may be little influenced by the experience as far as health symptoms are concerned. Moreover, it may be hypothesized that victimization is harmful to health only when the experience is linked to hurtful thoughts or feelings that may be present in loneliness. For peer victimized school children, loneliness may therefore be especially harmful. In addition, loneliness may be harmful among children without experiences of peer rejection or other peer harassment. This indicates that we need to be aware of loneliness at school – among all the children – and pedagogical practice that aims to promote inclusion and prevent loneliness should be highly acknowledged. The relation between loneliness, victimization, and children’s health needs to be further explored, also in longitudinal studies.

## Competing interests

The author declares that she has no competing interests.

## Author’s contributions

The present cross-sectional study is part of a two year follow-up, planned and administered by AL. The author designed the study, did the analyses, interpreted the data and wrote the paper.
